# Imaging and anatomical parameters of the lacrimal punctum and vertical canaliculus using optical coherence tomography

**DOI:** 10.7150/ijms.58291

**Published:** 2021-04-23

**Authors:** Jun Hu, Nan Xiang, Gui gang Li, Ban Luo, Yuan Zhang, Yingting Zhu, Rong Liu

**Affiliations:** 1 Department of Ophthalmology, Tongji Hospital, Tongji Medical College, Huazhong University of Science and Technology, Wuhan 430030, China.; 2 Tissue Tech, Inc., Ocular Surface Center, and Ocular Surface Research & Education Foundation, Miami, FL, 33173 USA.

**Keywords:** anatomical parameters, canaliculus, lacrimal punctum, optical coherence tomography, punctal lesions

## Abstract

**Purpose:** The anatomical parameters of normal lacrimal puncta and vertical canaliculus using optical coherence tomography (OCT) and the OCT imaging features of punctal lesions were analyzed to provide a basis for clinical diagnosis and treatment.

**Methods:** From June to September 2019, 40 volunteers (80 eyes) from Tongji Hospital were enrolled. The external punctal diameter (ELP) was measured using slit-lamp microscopy and OCT. The internal lacrimal punctal diameter (ILP) at 100 μm, vertical canalicular length (VCL), and tear meniscus depth were measured by OCT with open eyes. Twenty-eight volunteers (56 eyes) underwent the same examinations with their eyes closed. The OCT imaging features of 26 patients (27 eyes) with lacrimal lesions were examined.

**Results:** The ELP of the right and left healthy eyes under slit-lamp microscopy were 564.40 and 555.40 µm respectively. Under OCT, the ELP, ILP, and VCL of the right and left eyes were 628.20 um and 616.85 µm, 343.40 µm and 346.95 µm, 731.95 um and 709.20 µm respectively. The ELP was larger when measured by OCT than slit-lamp microscopy (p<0.05). Twenty-eight volunteers (56 eyes) had measurements taken under different conditions. The ELP, ILP, and VCL of the open and closed right eyes were 667.54 and 567.21 µm, 369.18 and 303.18 µm, 715.00 and 417.14 µm, respectively. The ELP, ILP, and VCL of the open and closed left eyes were 655.86 um and 551.68 µm, 369.25 um and 313.54 µm, 719.96 um and 433.89 µm respectively. The anatomical parameters of the open eyes were greater than those of the closed eyes (p<0.05). Thus, we identified the imaging features of lacrimal stenosis, punctal obstruction, punctal tear, lacrimal atresia, and lacrimal mass using OCT.

**Conclusions:** OCT can be used to measure the anatomical parameters of lacrimal puncta and vertical canaliculus *in vivo*. In addition, OCT can detect punctal lesions *in vivo* and provide an objective basis for the clinical diagnosis and treatment of punctal lesions.

## Introduction

The lacrimal punctum and canaliculus are important parts of the lacrimal drainage system, with approximately 80%-90% of tears draining into the nasal cavity through the inferior lacrimal punctum. Acquired or congenital abnormalities in the size and morphology of the lacrimal punctum and canaliculus may contribute to excess tears. The incidence of punctal stenosis is still unknown, with reported rates ranging from 8% to 54.3%, depending on the setting, demographics, and possibly interobserver variability 1. Most of the previous parameters of the lacrimal punctum and canaliculus were obtained from autopsies, without anatomical parameters *in vivo*. Slit-lamp microscopy is usually used to check the lacrimal punctum in clinical practice 1. Although easy to perform, this technique is highly subjective and lacks a unified standard for judging the size of the lacrimal punctum. In addition, it is difficult to observe the internal situation of the lacrimal punctum 1. Hurwitz et al. showed that ultrasound biomicroscopy (UBM) could be used to exam the lacrimal canaliculus 2. However, their study only provides limited data with low-resolution images, which is not conducive for observational decision-making 2. Furthermore, UBM has the drawback of requiring that the probe makes contact with the tissue 2. In contrast, optical coherence tomography (OCT) is a relatively new optical diagnostic technology which has a number of advantages. For example, it is painless, non-invasive and contactless, with a rapid image acquisition, a high-resolution and strong penetration 3. This evolving technology incorporates a shift from conventional time-domain OCT to spectral-domain OCT and the latest ultra-high-speed intuitive swept-source OCT. However, few studies have focused on the imaging of the punctum and canaliculus, and we currently lack anatomical parameters measured by OCT. Therefore, we aimed to examine the normal punctum and vertical canaliculus, measure their anatomical parameters using OCT and study the OCT imaging features of punctal lesions in order to provide an objective basis for their clinical diagnosis and treatment.

## Materials and methods

### Participants

This study was approved by the ethics committee of Tongji Hospital (Tongji Medical College, Huazhong University of Science and Technology) and adhered to the tenets of the Declaration of Helsinki. Informed consent was obtained from all participants. From June to September 2019, 40 healthy adults (22 male and 18 female), who volunteered to undergo OCT in Tongji Hospital affiliated to Tongji Medical College of Huazhong University of Science and Technology were enrolled. The inclusion criteria were as follows: age > 18 years, no symptoms of epiphora, normal morphology and structure of the punctum under slit-lamp microscopy, corneal fluorescence staining score 0, no history of eye surgery or trauma. In addition, 26 patients with lacrimal lesions who were treated in our hospital at the same time were also enrolled. The inclusion criteria were as follows: age > 18 years, symptoms of epiphora and abnormal punctum under slit-lamp microscopy.

### Image acquisition

All participants were examined using a slit-lamp microscope (ShangBangLS-6, Chong Qing) with their eyes open, and the magnification was adjusted to 16 X. With open eyes, the lower eyelid margin was gently everted approximately 60° using a cotton swab placed below the punctum just enough to get the punctum into a plane perpendicular to the light source. Images were acquired after the alignment was completed and analyzed using the slit-lamp microscope software measurement system.

All participants were examined using OCT (CASIA SS-1000, TOMEY, Japan). The SS-1000 OCT consists of a scanning source laser with a central wavelength of 1310 nm, providing higher sensitivity to imaging deep eye structures. It acquires 30,000 A-scans per second with a 10 μm axial resolution in tissue and a transverse resolution of 30 μm. A crossline scan (8 mm × 8 mm) was centered on the punctum for alignment. With the eyes open, the lower eyelid margin was gently everted approximately 60° using a cotton swab placed below the punctum just enough to get the punctum into a plane perpendicular to the light source. Images were acquired after the alignment was completed and analyzed using the SS-1000 OCT software measurement system.

The image sets of both lower lacrimal puncta were obtained by a single skilled operator. All subjects underwent a comprehensive ophthalmic examination, which included slit-lamp biomicroscopy and irrigation of the lacrimal passage to exclude undetected ocular pathology. During the examination, the subjects were allowed to blink naturally, and each eye was photographed three times. The clearest, deepest, and widest image was selected for measurement. During the process of everting the lower eyelid margin, movements were made gently, and excessive rollover was avoided of to prevent pulling and deformation of the punctum.

### Image analysis

**Figure 1:**
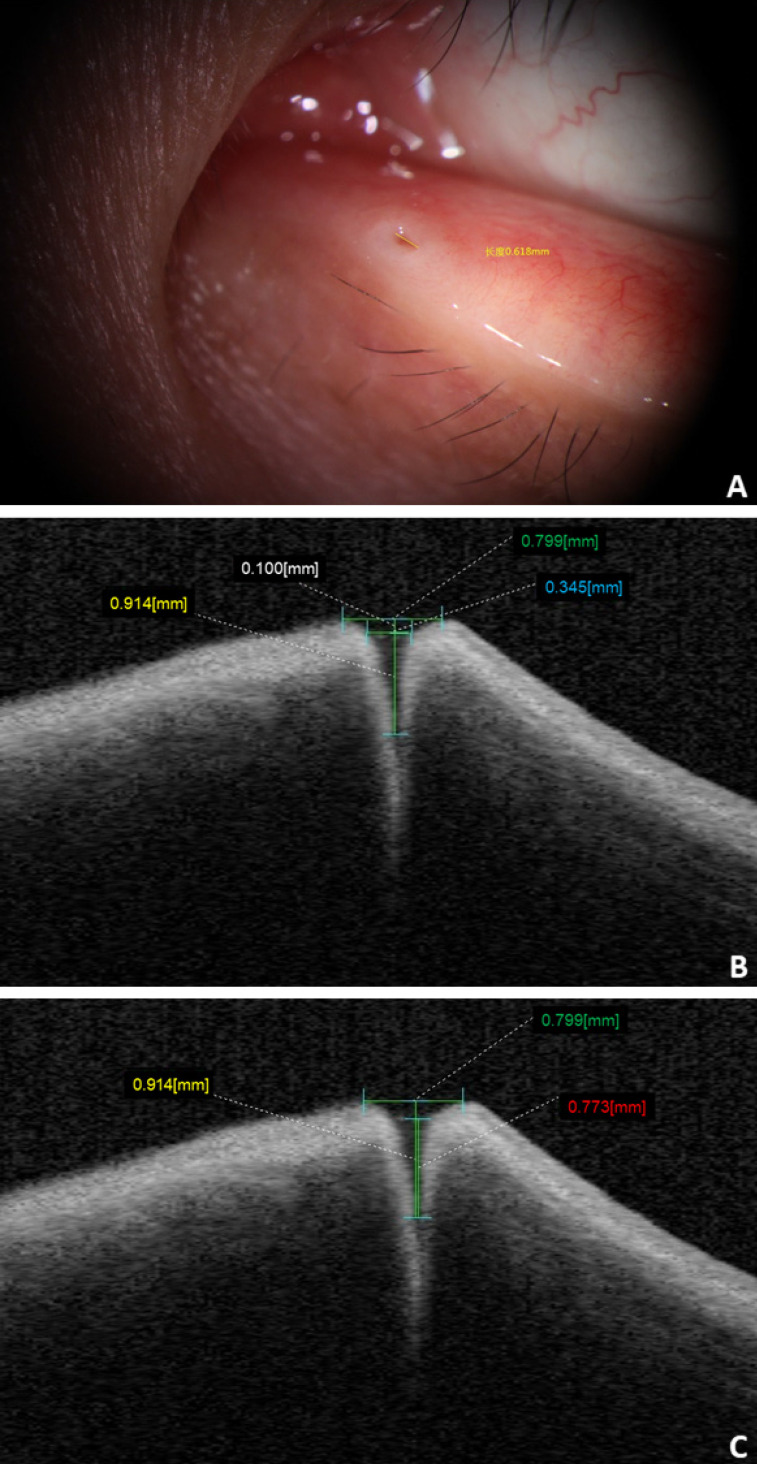
** A:** ELP under slit-lamp biomicroscope (yellow label). **B and C:** ELP (green label), ILP (blue label), VCL (yellow label), and tear meniscus depth (red label) from the OCT imaging.

The distance between the concave edge of the bilateral lacrimal papilla parallel to the eyelid margin was defined as the external punctal diameter (ELP) of the punctum under slit-lamp biomicroscopy. The ELP of the punctum under OCT was measured as a tangent connecting the highest points on the nasal and temporal punctal walls. The internal lacrimal punctal diameter (ILP) under OCT was measured at a depth of 100 μm. The vertical canalicular length (VCL) under OCT was measured as the height from the ELP tangent to the bottom of the vertical canaliculus. The tear well depth corresponds to the height from the surface of the tear well to the bottom of the vertical canalicular region (Fig. 1). In addition, we studied the OCT imaging features of punctal lesions.

### Statistical analysis

All data were analyzed using SPSS for Windows version 26 (SPSS Inc., Chicago, IL, USA). Normally distributed data are presented as mean and SD. A paired t-test was used to compare the numerical data of the left and right eyes and the open and closed eyes. An independent sample *t*-test was used to compare the numerical data of different genders, ELP under slit-lamp biomicroscopy and OCT, and ELP and ILP under OCT. Statistically significance was set at p < 0.05.

## Results

### Demographic characteristics

This study included 80 eyes of 40 volunteers, of whom 22 (44 eyes) and 18 (36 eyes) were male and female, respectively, with a mean age of 37.8 years (SD: 14.56 years; range: 19-73 years). Among the 28 volunteers (56 eyes) who underwent the examinations with their eyes open and closed, 15 (30 eyes) and 13 (26 eyes) were male and female, respectively, with a mean age of 37.5 years (SD: 14.72 years, range, 19-73 years). In addition, 26 patients (27 eyes) with lacrimal lesions were enrolled, including 12, 6, 5, 3, and 1 eye(s) with lacrimal stenosis, punctal obstruction, punctal tear, lacrimal atresia, and lacrimal mass, respectively.

### Normal OCT imaging

**Figure 2:**
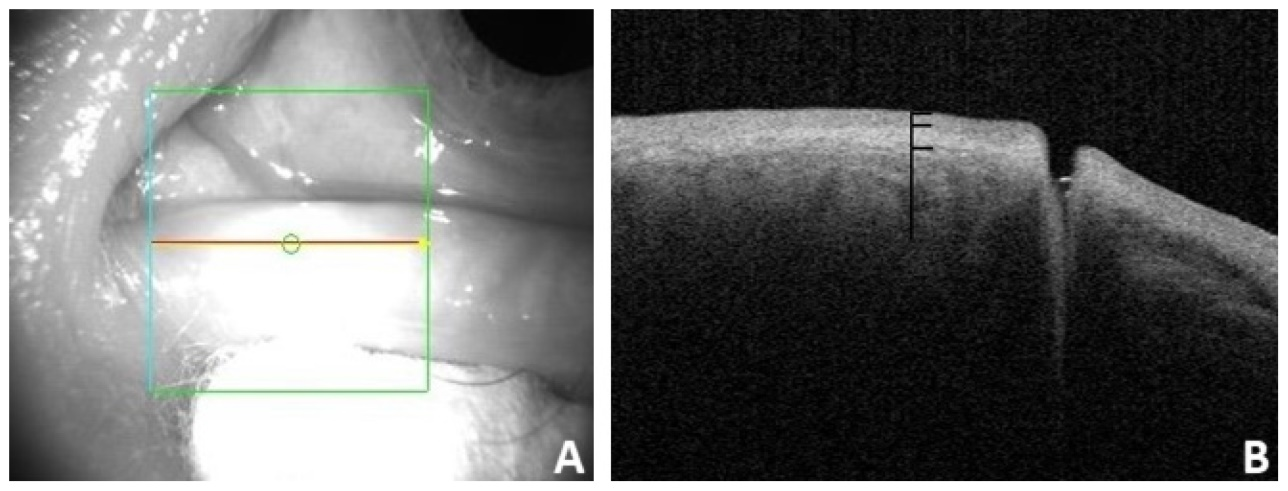
** A:** Appearance of the punctum under OCT. **B:** OCT imaging of the nasal and temporal palpebral margin tissue connected to the lacrimal papilla, showing a three-layer structure.

**Figure 3:**
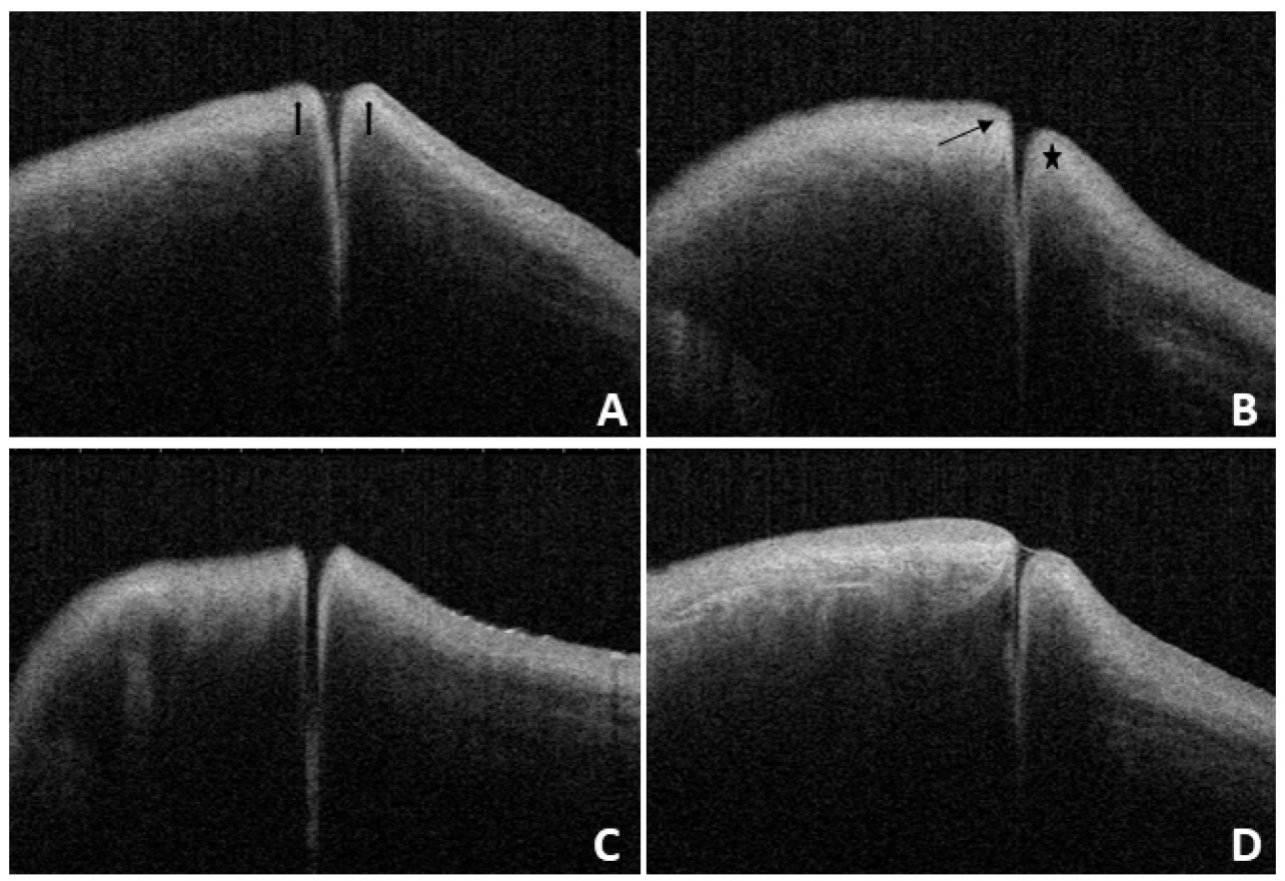
** A:** Under OCT, the nasal and temporal tissue of the punctum appears symmetrical (arrow). The overall morphology looks like a funnel and can be seen to gradually narrow.** B:** Under OCT, the nasal tissue is curved (star), while the temporal tissue is straighter (arrow). **C and D:** Different depth and size of the punctum and vertical canaliculus under OCT.

**Figure 4:**
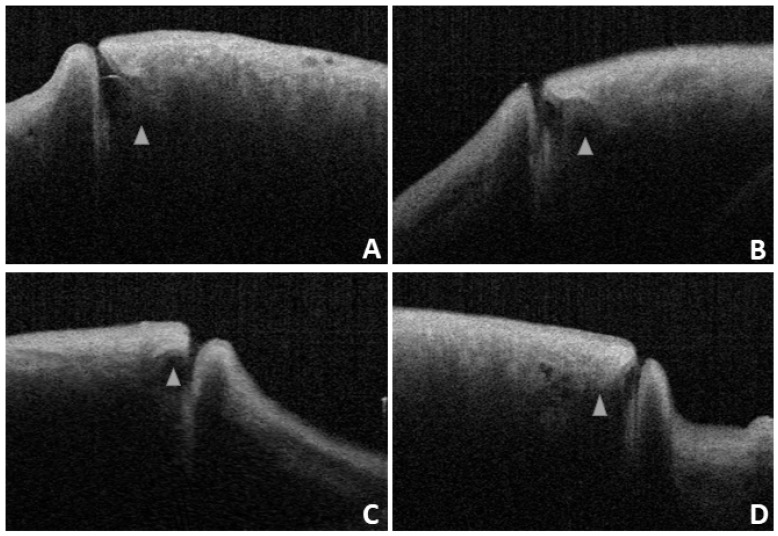
OCT images reveal a lacrimal ampulla/vertical canalicular dilatation in four puncta (**A and B** are left eyes, **C and D** are right eyes, and the triangle shows the ampulla).

In this study, we found that the OCT images of the nasal and temporal palpebral margin tissue connected to the lacrimal papilla displayed a three-layer structure, that is, a hyper-reflective layer sandwiched between two comparatively hypo-reflective layers (Fig. 2). Most OCT scans showed that the nasal tissue of the punctum was curved, while the temporal tissue of the punctum was straighter. In addition, some puncta were found to be symmetrical in the nasal and temporal tissues. The overall morphology of the punctum and vertical canaliculus gradually narrowed. When a tear remains in the punctum and vertical canaliculus, the tear plane can be observed on OCT (Fig. 3). Significant dilation of the vertical canaliculus, called an “ampulla”, was visible in four punctal systems and was mostly toward the temporal side of the punctum (Fig. 4).

### Normal anatomical parameter

**Figure 5:**
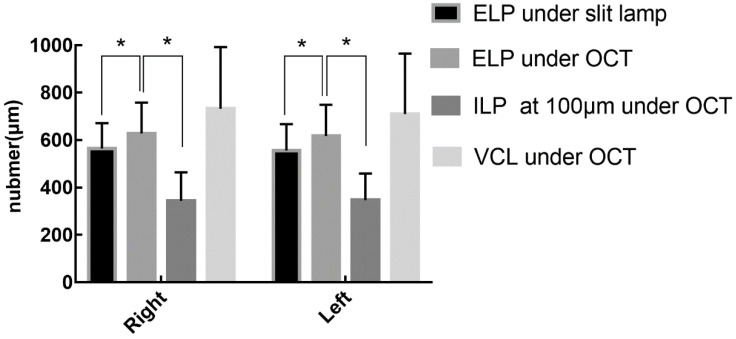
There were statistically significant differences between the ELP under OCT and slit-lamp microscopy and between the ELP and ILP under OCT.

**Figure 6:**
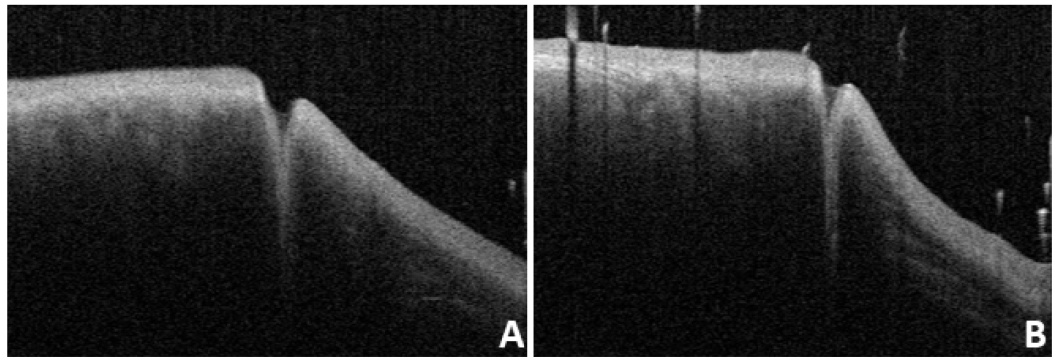
** A and B:** OCT images of the same subjest with the eyes open (A) and closed (B).

**Figure 7:**
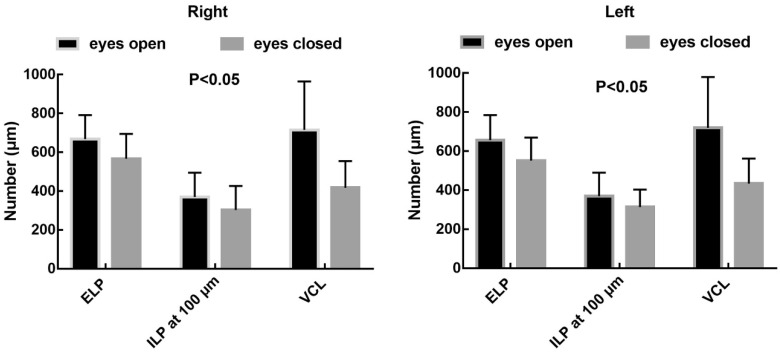
There were statistically significant differences between ELP, ILP, and VCL when the eyes were open or closed.

Anatomical parameters under slit-lamp biomicroscopy and OCT in healthy adults with their eyes open are shown in Table 1. The ELP measured by OCT was larger than that measured by slit-lamp microscopy, and the difference was statistically significant (p<0.05). The ELP was larger than the ILP under OCT, and the difference was statistically significant (p<0.05; Fig. 5). The VCL of the right and left eyes were 731.95 μm (SD: 260.26) and 709.20 μm (SD: 254.97), respectively. Tear residues were found in 66.3% of puncta, and the tear meniscus depths of the right and left eyes were 584.31 μm (SD: 215.72) and 568.50 μm (SD: 235.78), respectively. There were no statistically significant differences in the anatomical parameters of the punctum and vertical canaliculus between men and women and between the left and right eyes (p>0.05; Table 2). The anatomical parameters of 28 subjects (56 eyes) with their eyes open and closed are shown in Table 3. When the eyes were closed, the ELP, ILP, and VCL were smaller than they were when the eyes were open, and the differences were statistically significant (p<0.05; Fig. 6, 7).

**Figure 8:**
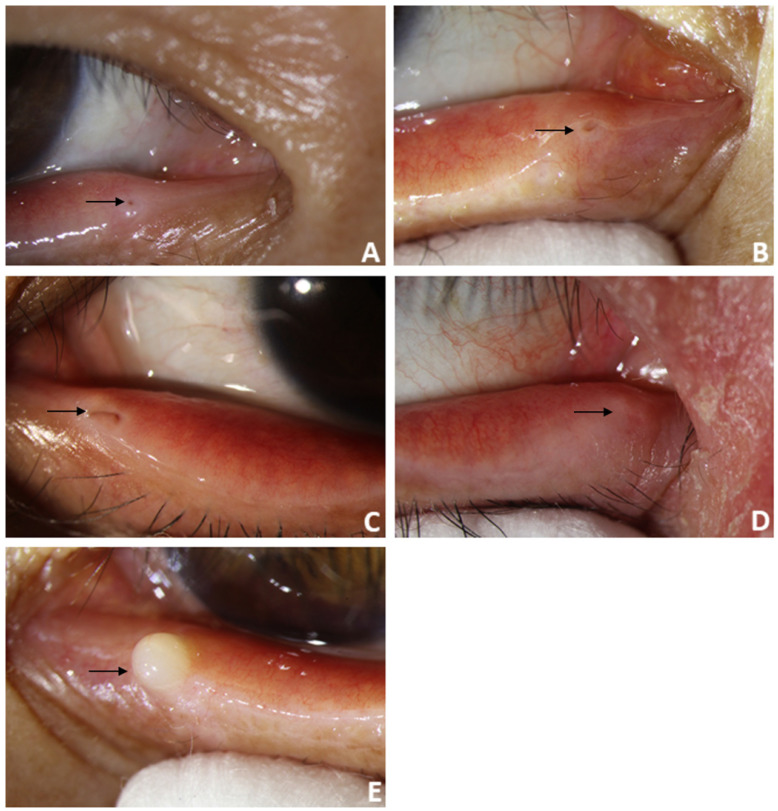
Slit-lamp microscope image of lacrimal lesions. **A:** punctal stenosis, **B:** punctal obstruction, **C:** punctal tear, **D:** punctal atresia, **E:** punctal mass.

**Figure 9:**
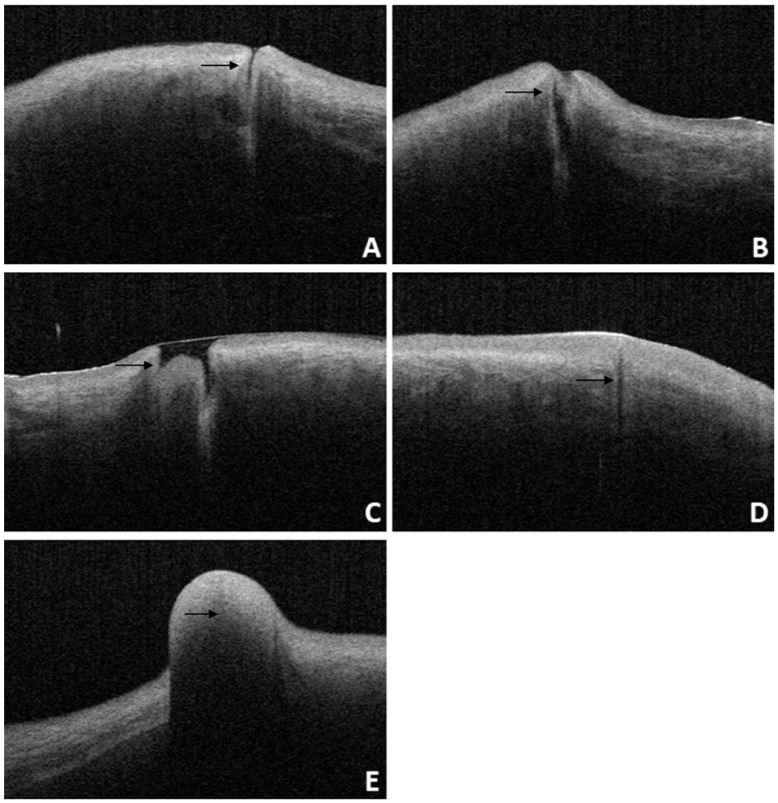
OCT image of lacrimal lesions. **A:** punctal stenosis, **B:** punctal obstruction, **C:** punctal tear, **D:** punctal atresia, **E:** punctal mass.

For patients diagnosed with punctal stenosis, OCT showed that the opening of the lacrimal punctum was narrow and small, and the lumen of the vertical canaliculus looked like a fissure. In the case of patients diagnosed with punctal obstruction, OCT showed that the opening of the lacrimal punctum was shielded by a banded membrane-like material, one end was connected to the lacrimal wall, while the other was suspended near the opening of the punctum. Furthermore, the vertical canaliculus was visible. In patients diagnosed with punctal tear, the lacrimal punctum had an abnormal shape, and the opening was larger than normal. In cases of punctal atresia, punctal openings were not observed in either eye, while the lumens of the inferior vertical canaliculi were visible in two eyes. Furthermore, the lacrimal punctum and vertical canaliculus were not observed in one eye. For the patient with a mass in the punctum, OCT showed a bulge with a high and medium reflection on the lacrimal punctum without a normal punctum and vertical canalicular structure (Fig. 8, 9).

## Discussion

OCT is a newly developed optical diagnostic technique that was employed for the first time by Izatt for imaging the anterior segment in 1994 4. OCT has a number of advantages, such as being non-invasive, painless andcontactless, achievinghigh-resolution. Therefore, it has been widely used in clinical practice. In recent years, OCT has been applied to the examination of the proximal lacrimal system, and studies have attempted to measure the anatomical parameters, evaluate the effect of punctoplasty, and examine lacrimal lesions 5-7. Wawrzynski et al. found that the mean outer punctual diameter under OCT was 247 μm (SD: 78) 7. However, the specific measurement range of the outer punctual diameter and the ILP under OCT was not specified. Timlin et al. reported that the mean outer punctual diameter was 646 μm (SD: 150), and the mean inner punctual diameter at a depth of 100 μm was 257 μm (SD: 123) 6. Liquid was detected in 73% of the puncta, and the mean depth of the fluid level was 192 μm (SD: 207) 6. Tao Hai et al. reported that the mean outer punctual diameter was 548.4 μm (SD: 130.5), and the mean inner punctual diameter at a depth of 100 μm was 262.8 μm (SD: 120.8) by OCT 8. The mean outer punctal diameter measured by Timlin et al. was similar to the ELP in the current study, but their results were higher than those of other researchers, such as Wawrzynski. It is speculated that this may be because different researchers have different definitions of the measurement range of the punctum. Second, different OCT types and the ethnicity of the subjects could lead to different measurements; however, few studies have examined the effect of ethnic differences on punctual diameter. Currently, there is no unified standard for the measurement range of the outer punctual diameter.

At present, it is generally believed that the vertical canaliculus measures approximately 2 mm. This structure consists of two parts: the vertical part and the ampulla. The vertical part is formed by the downward continuation of the punctum, which accounts for approximately 1/4th of the total length of the vertical canaliculus. The ampulla is a significant dilation of the vertical canaliculus, connected with the horizontal canaliculus on the nasal side. Timlin et al. identified an ampulla in three of 40 eyes and found that they generally occur below the lateral part of the punctum. They speculated that due to the collapse of part of the vertical canaliculus, it was challenging to find the lower ampulla under OCT 6. In this study, a significant dilation of the vertical canaliculus was visible in the four punctal systems. Wawrzynski et al. reported that the VCL ranged from 392 μm to 1242 μm (mean: 753, SD: 216) 7. The depth measured in Kamal's study was 890.41 μm (SD: 154.76) compared to Timlin et al., who reported a maximum detected depth of 1308 μm (mean: 544, SD: 327) 6, 9. The maximum detected depth was significantly shorter than the anatomically stated length of 2 mm. It has been speculated that the influence of dynamicity of the eyelids, penetration depth of the light source, differences between ethnicities, tissue fixation embalming in cadavers, the collapse of part of the vertical canaliculus, and the flipping and stretching during measurement might lead to differences between OCT and autopsy measurements 7, 9-12. It is believed that the depth of the vertical canaliculus is approximately 1 mm 13. However, the true depth of the vertical canaliculus remains to be elucidated due to lack of autopsy studies on this topic. Allam et al. found that the right and left sides showed no difference in all the measured parameters 12. In the current study, the anatomical parameters of the punctum and vertical canaliculus between the left and right eyes also showed no difference, and we found no significant differences in the anatomical parameters of the punctum and vertical canaliculus between the sexes.

The characteristic presence of dense fibrous tissue in continuity with the tarsal plate in and around the punctum was histologically demonstrated by Kakizaki et al 14. Timlin et al. found that OCT imaging of the palpebral margin tissue of the nasal and temporal punctum showed three layers and speculated that the highly reflective layer observed on OCT corresponded to dense fibrous tissue 6. Scanning electron microscopy of the normal lacrimal canaliculus found multiple collagen fibers attached to the muscle bundles of the orbicularis oculi on the outer surface of the distal lacrimal canaliculus, indicating that this muscle might play a potential role in tear drainage 15. The orbicularis oculi muscle and lacrimal sacs can be compressed and relaxed through blinking to form a pump-like mechanism that drains tears into the nasal cavity 16-18. When the eyes are closed, the punctum and orbicularis oculi muscle around the lacrimal canaliculus contract, causing the punctum to shrink and move inward. Meanwhile, the lacrimal canaliculus shortens and is compressed, together with the lacrimal sac, enabling tears to flow from the nasolacrimal duct into the nasal cavity. When the eyes are open, the punctum and orbicularis oculi muscle around the lacrimal canaliculus dilate, causing the punctum to reopen, and the lacrimal canaliculus and lacrimal sac to expand and extend. Meanwhile, the vertical canaliculus dilates to form an ampulla, enabling tears to enter the lacrimal canaliculus and lacrimal sac by siphoning. In this study, 28 patients (56 eyes) underwent examinations with their eyes open and closed. When the eyes were closed, ELP, ILP, and VCL were smaller than when the eyes were open, and the differences were statistically significant (p<0.05; Table 3 and Fig. 7). The results of this study are consistent with the theory of the lacrimal pump, providing a certain degree of objective imaging basis for the mechanism of the lacrimal pump.

OCT also plays an important role in the evaluation of punctoplasty and the diagnosis and treatment of punctal lesions such as punctal stenosis, punctal obstruction, punctal tear, punctal atresia, and punctal mass. Timlin et al. performed preoperative OCT scanning on 20 patients with epiphora as well as on 20 healthy volunteers and found that those patients with improved epiphora had a significantly smaller preoperative punctal diameter at a depth of 100 μm on OCT compared with healthy controls 19. However, there was no significant difference in the diameter of the punctum at the entrance of the punctum or at a depth of 500 μm 19. In 2018, Timlin et al. found that OCT can help detect the presence of the lacrimal canaliculus in patients with punctal atresia or absence, as well as predict the possibility of successful reduction during surgical exploration 20. For patients diagnosed with punctal atresia, our study found that punctal openings were not observed in either eyes. At the same time, the lumens of the inferior vertical canaliculi were visible, and the lacrimal punctum and vertical canaliculus were not visible in one eye. The intraoperative exploration results were consistent with those of the OCT examination. OCT will also have great application value in the future. Accurate imaging and measurement of punctal size using OCT will play an important role in the diagnosis of punctal stenosis and the guidance of the diagnosis and treatment of clinical punctal diseases. Customized punctal plugs can be used to treat patients with severe dry eye disease. OCT could also be utilized to monitor the effect of surgery or drugs on punctal size, which could be used to study the physiological functions and mechanisms of the proximal lacrimal system in the future.

This study has several limitations. Firstly, the eyelid margin needs to be everted to expose the lacrimal punctum, which may cause artificial error. Therefore, the measurement should be performed by a practician. Secondly, the age span of the volunteers was 19-73 years, because of the relatively small number of subjects included in the current study, a larger sample size with different age groups is required to establish the normal range of OCT anatomical parameter values in different age groups.

In conclusion, OCT can be used to measure the anatomical parameters of the lacrimal puncta and the vertical canaliculus *in vivo*. The anatomical parameter measurements when the eye is open and closed provide objective evidence for the lacrimal pump mechanism to some extent. OCT of punctal lesions can provide an objective basis for clinical diagnosis and treatment.

## Figures and Tables

**Table 1:** Anatomical parameters of the right and left punctum and vertical canaliculus

Anatomical parameter	Right (μm)	Left (μm)	*t*-value	*p*-value
ELP under slit-lamp microscopy	564.40 (107.25)	555.40 (111.83)	1.398	0.170
ELP under OCT	628.20 (129.36)	616.85 (131.81)	1.270	0.212
ILP under OCT	343.40 (120.58)	346.95 (112.20)	-0.038	0.760
VCL under OCT	731.95 (260.26)	709.20 (254.97)	0.984	0.331

**Table 2:** Anatomical parameters of the punctum and vertical canaliculus in males and females

Anatomical parameter	Right (μm)				Left (μm)			
	Male (n=22)	Female (n=18)	*t-*value	*p-*value	Male (n=22)	Female (n=18)	*t-*value	*p-*value
ELP under slit-lamp microscopy	566.59 (120.14)	561.72 (92.45)	0.141	0.889	556.50 (107.99)	554.06 (119.51)	0.068	0.946
ELP under OCT	649.27 (148.36)	602.44 (99.23)	1.189	0.242	635.77 (139.32)	593.72 (121.86)	1.004	0.322
ILP under OCT	354.55 (140.16)	329.78 (93.44)	0.641	0.525	355.55 (119.88)	336.44 (104.48)	0.531	0.599
VCL under OCT	746.68 (292.63)	713.94 (221.31)	0.391	0.698	739.86 (306.06)	671.72 (175.26)	0.882	0.384

**Table 3:** The anatomical parameters under OCT with the eyes open and closed

Anatomical parameter	Right (μm)				Left (μm)			
	Eyes open (μm)	Eyes closed (μm)	*t-*value	*p-*value	Eyes open (μm)	Eyes closed (μm)	*t-*value	*p-*value
ELP under OCT	667.54 (123.60)	567.21 (127.29)	8.657	0.000	655.86 (128.49)	551.68 (118.18)	9.702	0.000
ILP under OCT	369.18 (125.12)	303.18 (122.70)	4.918	0.000	369.25 (120.15)	313.54 (89.46)	3.571	0.001
VCL under OCT	715.00 (249.33)	417.14 (137.52)	6.839	0.000	719.96 (259.77)	433.89 (127.96)	6.511	0.000
